# Zeolite Supported Pt for Depolymerization of Polyethylene by Induction Heating

**DOI:** 10.1021/acs.iecr.2c04568

**Published:** 2023-05-24

**Authors:** Bernard Whajah, Joseph N. Heil, Cameron L. Roman, James A. Dorman, Kerry M. Dooley

**Affiliations:** † Cain Department of Chemical Engineering, Louisiana State University, Baton Rouge, Louisiana 70803, United States; ‡ Department of Chemistry and Physics, LeTourneau University, Longview, Texas 75602, United States

## Abstract

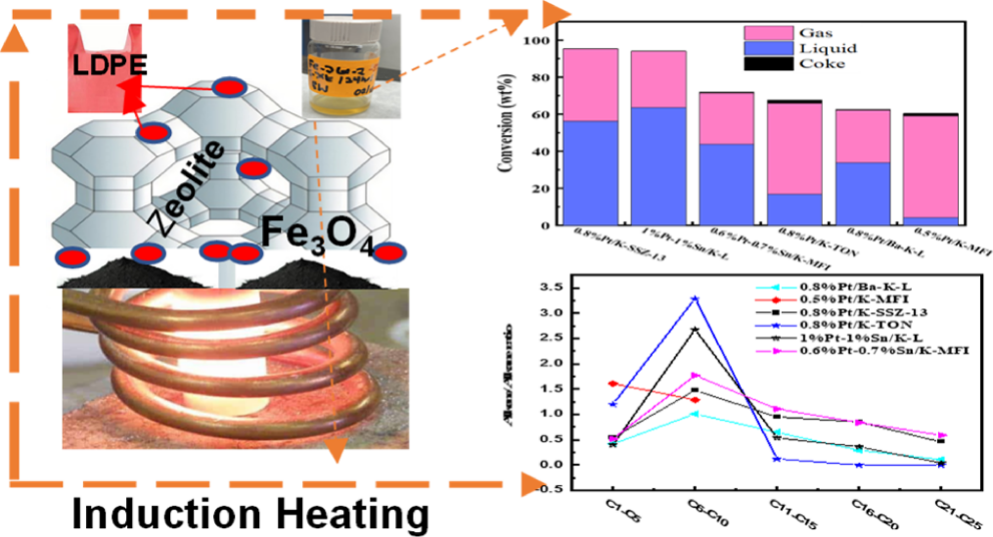

We demonstrate that for polyethylene depolymerization with induction heating (IH), using a bifunctional (Pt- or Pt–Sn-containing zeolite) hydrocracking catalyst, we can obtain high hydrocarbon product yields (up to 95 wt % in 2 h) at a relatively low surface temperature (375 °C) and with a tunable product distribution ranging from light gas products to gasoline- to diesel-range hydrocarbons. Four zeolite types [MFI, LTL, CHA(SSZ-13), and TON] were chosen as the supports due to their varying pore sizes and structures. These depolymerization results are obtained at atmospheric pressure and without the use of H_2_ and result in an alkane/alkene mixture with virtually no methane, aromatics, or coke formation. We also demonstrate how IH helps overcome diffusional resistances associated with conventional thermal heating and thereby shortens reaction times.

## Introduction

The unique physical and chemical properties of plastics and their affordability make them indispensable, and their production rates have increased exponentially. Since the 1950s, plastic production has risen by ∼6 million metric tons (MMt) annually to 110 MMt (∼75 lbs of plastic per person per year).^1^ Of the plastics produced, only 7% have been recycled, with the remaining waste plastics being discarded or incinerated.^2^ Additionally, plastics production is expected to claim 15% of the annual greenhouse gas allotments necessary to limit the global temperature increase to 1.5 °C.^2^

Polyethylene (PE) constitutes about 36% of all plastics produced globally.^3^ While the strong and stable aliphatic C–H and sp^3^ C–C bonds in PE contribute to its desirable mechanical and chemical properties, these same bonds make it challenging to depolymerize and repurpose. Therefore, the greater part of end-of-life PE is landfilled. Bardow et al. have claimed that a net-zero plastics economy is possible with potential cost savings of $288 billion.^4^ However, their lifecycle analysis presupposes current plastics reprocessing technologies, specifically pyrolysis to naphtha followed by steam or high-pressure hydrocracking, both of which are energy-intensive, not viable under current economic conditions, and have been identified by the Department of Energy as “insufficient to address the growing accumulation” of plastic waste.^5^ The ceiling temperature of PE is roughly 367 °C,^6^ which necessitates using a reactive gas such as hydrogen or, more recently, ethylene^7^ to depolymerize PE under milder conditions. Catalytic hydrogenolysis has gained much attention in recent reports as a method to upcycle PE to valuable alkanes at lower temperatures with a narrower product distribution than is possible by either catalytic or non-catalytic pyrolysis.^3,8−10^

Obtaining significant conversion and selectivity to liquid alkanes via hydrogenolysis with most oxide-supported transition metal catalysts requires long reaction times (6–96 h) and high H_2_ pressures (1.2–18 MPa).^3,8,10−12^ Over 5 wt % Ru/C, a 45 wt % yield of C8–C45 *n*-alkanes was obtained from PE at 20 bar H_2_ pressure, 200 °C for 16 h.^8^ At 250 °C, 16 h, and 16 bar H_2_ pressure, 60–80 wt % lubricant hydrocarbons were produced from polypropylene using Ru/TiO_2_.^13^ Celik et al.^12^ used Pt-decorated SrTiO_3_ to completely convert PE to generate lubricant and wax-type hydrocarbons (280 °C, 96 h, 11.7 bar H_2_). Hydrogenolysis in conjunction with aromatization to make long-chain alkylaromatics (>50% on a carbon basis) occurs when Pt/Al_2_O_3_ is used as the catalyst (280 °C and 24 h)^9^ while dehydrogenation (200 °C) and tandem isomerization and ethenolysis (130 °C, 16 h, and 25 bar ethylene) gave >80% yields of propylene from high-density polyethylene (HDPE) with *p*-xylene as the solvent.^7^

Hydrocracking of polyolefins using bifunctional catalysts (metal/acid) has been shown to take place at lower temperatures with relatively shorter reaction times and narrower product distributions compared to pyrolysis.^11^ Metals such as Pt, Pd, Ni, or Co, all known to possess dehydrogenation–hydrogenation activity, can be incorporated into acidic zeolites to obtain such catalysts.^10,14−19^ At 250 °C, 2 h, and 30 bar H_2_, a mixture of gasoline, diesel, and jet fuel range hydrocarbons with a maximum yield of 85 wt % was obtained over a physical mixture of Pt/WO_3_/ZrO_2_ and H–Y zeolite.^11^ Impregnating Pt into USY and BEA zeolites, Bin Jumah et al. similarly generated ca. 95 wt % hydrocarbon oil at 330 °C and 20 bar H_2_.^20^

Promotion of Pt by Sn in the dehydrogenation of light alkanes to alkenes often increases long-term catalyst activity and decreases the production of cracking products and coke, presumably by breaking up larger Pt ensembles and covering sites of high coordinative unsaturation (“geometric” effects), also modifying the electronic properties of small Pt clusters.^16,17,21−27^ In addition, the acid content and strength, porosity, and morphology of the zeolite type are expected to influence the size and shape selectivity of the depolymerization products. Larger pore size zeolites are expected to give relatively heavier products.

Microwave heating has been explored as an alternative to thermal heating in the depolymerization of polymers since the electromagnetic radiation can directly interact with the polymer and catalyst.^28,29^ With a 1:1 FeAlO_*x*_/HDPE ratio, Jie et al. reacted HDPE using microwave heating to obtain gas yields of ∼65 wt %, but most of the remaining product was carbon, either as coke or iron carbide.^30^ The microwave-based process requires a solvent to prevent runaway catalyst heating and localized pyrolysis,^31,32^ which caused the carbon formation.^30^

Recently, our group has employed induction heating (IH) methods to break down plastics over dual-functional Fe_3_O_4_ and Ni- or Pt-based zeolite and metal oxide catalysts.^33^ We generated high concentrations of alkene/alkane hydrocarbons with relatively narrow product distributions.^33^ An IH-driven process offers several benefits over traditional thermal heating routes,^34,35^ in particular a large interface/bulk temperature gradient (so only the polymer in the vicinity of the catalyst particles reaches reaction temperatures) and the apparent inhibition of some secondary reactions in that low amounts of methane, aromatics, and coke are produced. At a surface temperature of 420 °C, with a catalyst-to-polymer ratio of 1:10 and no added H_2_, a maximum conversion of 82 wt % was obtained in 2 h with 0.5 wt % Pt/K-MFI.^33^ However, the liquid products only accounted for 2% of the total conversion by weight, the remaining being mostly gaseous products with 0.3 wt % coke formation.

Herein we demonstrate that for PE depolymerization with IH, using a bifunctional (Pt- or Pt–Sn-containing zeolite) hydrocracking catalyst and varying the type of zeolite, we can obtain even higher hydrocarbon product yields (up to 95 wt % in 2 h) at a relatively lower surface temperature (375 °C) and with a tunable product distribution ranging from light gas products to gasoline- to diesel-range hydrocarbons. Four zeolite types [MFI, LTL, CHA(SSZ-13), and TON] were chosen as the supports due to their varying pore sizes and structures. These depolymerization results are obtained at atmospheric pressure and without the use of H_2_. The goal of this work is to understand how Pt, with or without Sn, on zeolite supports differing in pore geometry can affect product selectivity, activity, and coke formation in the depolymerization of PE using IH. We also demonstrate how IH helps overcome diffusional resistances associated with conventional thermal heating and thereby shortens reaction times.

## Methods

### Catalyst Synthesis

The zeolites (MFI, LTL, TON, and SSZ-13 structure types, further details in the Supporting Information, and Figure S1b–e) were ion-exchanged to the K^+^ form twice with excess 0.1 M KOH at 80 °C [or with a mixture of KOH and 0.1 M Ba(NO_3_) for Ba-K-L], dried at 120 °C overnight, then at 350 °C in flowing air. The Pt was added to K^+^-exchanged zeolites (Pt/K-TON, Pt/K-SSZ-13, Pt/K-MFI, and Pt/Ba-K-L) by contacting overnight with dilute aqueous platinum diaminodinitrite (PtH_4_N_4_O_4_, Matthey-Bishop, 99.99%) at pH = 10. The solution was slowly evaporated at 120 °C, dried overnight, and then reduced with 5% H_2_ (Airgas, Certified) at 500 °C for 6 h. For PtSn catalysts, the desired amount of tetrabutyltin (Aldrich, 93%) was dissolved in pentane, and the zeolite was impregnated under N_2_, dried under N_2_ at 120 °C for 3 h, and then impregnated with Pt in the same way as the Pt-only catalysts. These were calcined in 30% O_2_/Ar at 300 °C for 3 h, then pulse reduced with H_2_ (at 400 °C, PtSn/K-MFI, PtSn/K-L). Commercial Fe_3_O_4_ (Alfa Aesar, 97%, 50–100 nm diameter) nanoparticles were used.

### RF-Activation Reaction Experiments

A total of 200 mg of the catalyst/Fe_3_O_4_ powder (1:1 wt ratio) mixture were added to 1 g of low-density polyethylene (LDPE) polymer (Alfa, 924 kg/m^3^, melting point 105–115 °C). The mixture was loaded into a glass reactor (Figure S1a), purged with N_2_, and either exposed to the RF field from an Ambrell EASYHEAT 8130LI 10 kW induction heater with a 3-turn, 0.035 m diameter Cu coil (200–500 A, 22–54 mT equivalent), or immersed in a heated sand bath, in both cases for 2 h at near atmospheric pressure. The reactor was cooled for 30 min before collection of gas/liquid products. The surface temperature–RF amperage calibration was taken from previous work.^33^ A 500 A RF input gives roughly a 375 °C surface temperature, and the corresponding temperatures for other currents are: 400 A, 340 °C; 300 A, 285 °C; 200 A, 122 °C.

### Product Analysis

The gas atmosphere was sampled during the experiment and analyzed by injection into an SRS RGA200 residual gas analyzer operating in selective ion mode at the parent and other key *m*/*z* values. The algorithm to convert from pressure differences at specific *m*/*z* to gas mol fractions is given in the Supporting Information. Pressure-ion count calibration factors were determined by the injection of standards. Other (liquid) depolymerization products were extracted from the remaining polymer/catalyst mixture with dichloromethane for 5 d. The liquid products were then analyzed by gas chromatography–mass spectrometry on an Agilent 6890 (100 m × 0.25 mm SPB-1 column). The conversion to gases was determined from the weight change before and after the reaction. The conversion to liquids was determined from the weight change upon drying a sample of catalyst/product mass under vacuum at 190 °C for 4 d. Coke amounts were determined by temperature-programed oxidation (TPO) in air, 50–250 °C, 10 °C/min, hold 60 min, 10 °C/min to 420 °C, hold 40 min, and 10 °C/min to 650 °C, hold 60 min. The combined gas, liquid, and coke analyses, along with the conversions, were combined to determine mol %’s of all products on a combined basis, taking coke as a CH polymer. The product selectivity (*S*_i_) is then defined as
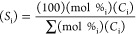
1where *C*_i_ is the number of carbons in the compound.

### Catalyst Characterization

Surface areas were determined by the Brunauer–Emmett–Teller method (Micromeritics ASAP 2020). Pore volumes were determined from the N_2_ adsorption branch at *P*/*P*_0_ = 0.99. Micropore volumes were computed by the *t*-plot method using the Harkins–Jura correlation. The Pt dispersions were determined by pulse chemisorption of H_2_ on a Micromeritics 2700 at 23 °C (assumed 1 H/Pt, see eq S1 in the Supporting Information). Thermogravimetric analysis/differential scanning calorimetry (TA SDT 6000) of 1-propylamine (1-PA) was employed to titrate the Brønsted sites, as discussed by Gorte^36,37^ and Price and Dooley.^38^ Powder X-ray diffraction (XRD) data were obtained using Cu Kα1 (λ = 1.54 Å) emission on a PANalytical XRD with a step size of 0.04° and a dwell time of 60 s.

X-ray absorption spectroscopy measurements were conducted using the WDCM 2.0 beamline of the electron storage ring at the LSU Center for Advanced Microstructures and Devices (CAMD). Measurements were done at the Pt L_III_-edge in fluorescence mode with a Si(111) crystal monochromator. The beamline was calibrated with Pt foil (*E*_0_ = 11 564 eV). Integration time was adjusted to obtain adequate counts up to *k* = 10 Å^–1^. Runs were repeated three times. Background subtraction, deglitching, and merging of spectra were done using Athena 0.9.061. Pt L_III_-edge XAFS fitting was performed in Artemis 0.9.26. Three parameters were varied to obtain the best possible fits of the data to various bulk structures, σ^2^ (Debye–Waller factor), Δ*E*_0_ (deviation in *E*_0_ caused by structural deviations from the ideal crystal structure), and Δ*R* (deviation in the interatomic distance). A Pt foil standard was fitted first to obtain *S*_0_^2^ (the amplitude reduction factor), with known coordination numbers and Δ*R*’s. The fitting range in *R* space was 1–5 Å, and all significant scattering paths as identified by Artemis were included.

X-ray photoelectron spectroscopy (XPS) data were collected using a Scienta Omicron ESCA 2SR XPS equipped with a monochromatic Al Kα (*h*ν = 1486.7 eV) X-ray source and a hemispherical analyzer with a 128-channel detector at 1.5 × 10^–9^ Torr. The Gaussian width of the photon source was 0.2 eV. The adventitious carbon C 1s peak at 284.8 eV was used to calibrate the energies. After Shirley background subtraction, all peaks were fitted using Casa XPS (version 2.3.25) as asymmetric Lorentzians (Pt) or Gaussian–Lorentzian (Sn, Al).

Morphology and Pt/zeolite crystal particle sizes were analyzed at the Oak Ridge National Laboratory by high-resolution transmission electron microscopy (HRTEM) using a 200 kV JEOL NEARM electron microscope equipped with double aberration correctors, a dual-energy-loss spectrometer, and a cold FEG source. Before imaging, the samples were dispersed in ethanol and drop-cast on a 300 mesh, lacey carbon grid.

## Results and Discussion

### Properties and Surface Characterization of Unused Catalysts

Some chemical and physical properties of the unused catalysts are given in Table 1. The N_2_ adsorption–desorption isotherms are given in Figure S3. As expected, the addition of Sn decreased the Pt dispersion (H/Pt) of K-L, indicating Pt’s ability to alloy with Sn even in confined pore spaces.^17,39−42^ But this was not the case for the MFI zeolite. As shown by Corma and co-workers, the reduction of low-loading Pt–Sn in MFI can result in highly dispersed Pt(0) clusters with little to no direct contact with Sn^2+^, and the computed Pt dispersions can be high.^15^ The measured dispersion in Table 1 is in line with Corma’s results.

**Table 1:** Properties of Catalysts Used for Depolymerization of LDPE

catalyst	Si/Al	Pt loading (wt %)	Sn loading (wt %)	surface area (m^2^/g)	pore volume (cm^3^/g)	micropore volume (cm^3^/g)	Pt dispersion (%)^a^
Pt/K-MFI	29	0.5		370	0.25	0.11	34
PtSn/K-MFI	29	0.5	0.7	300	0.18	0.12	65
Pt/Ba-K-L	9	0.8		190	0.2	0.08	90
PtSn/K-L	9	1	1	180	0.19	0.07	12
Pt/K-TON	55	0.8		53	0.11	0.02	31
Pt/K-SSZ-13	16	0.8		250	0.27	0.08	93
Sn/K-MFI^b^	29		7.3				
Fe_3_O_4_^b^				33	0.11		

a The equation for the calculation is shown as eq S1.

b Did not adsorb H_2_ at 23 °C.

**Figure 1:**
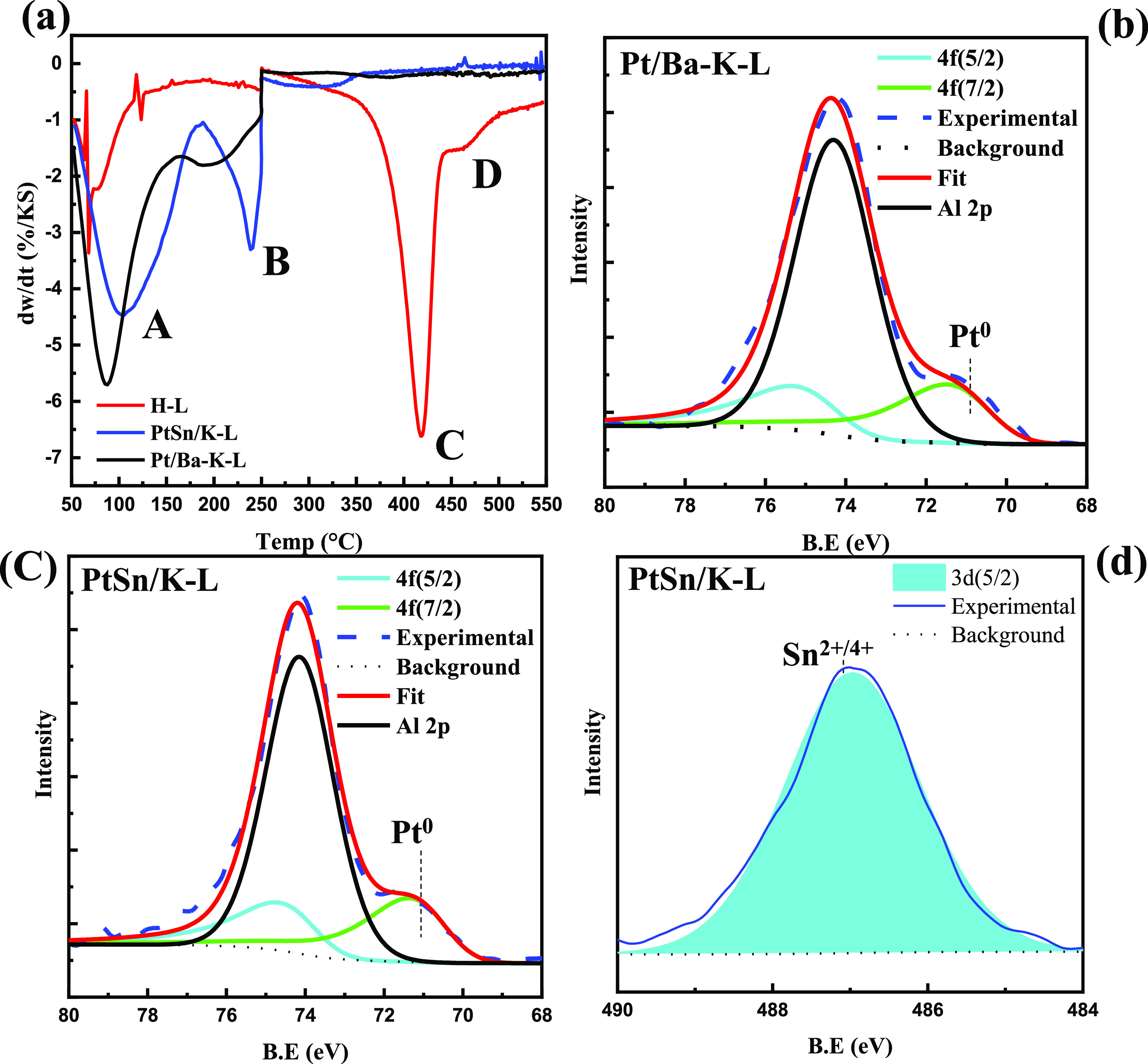
(a) Desorption of 1-PA from LTL zeolites. Samples saturated with 1-PA at 50 °C prior to TPD. XPS spectra of: (b) Pt/Ba-K-L, Pt 4f_5/2,7/2_; (c) PtSn/K-L, Pt 4f_5/2,7/2_; and (d) PtSn/K-L, Sn 3d_5/2_.

Earlier work had established that ion-exchange of the zeolites to replace H^+^ with K^+^, prior to the introduction of the active metals (either Pt or Pt/Sn), gave more active depolymerization catalysts that were also less prone to coke formation.^33^ After exchanging K^+^ with the active metals, the Brønsted/Lewis acid site concentrations and strengths were quantified using 1-PA desorption. Peak temperature shifts and decreases in adsorbed amounts are associated with the replacement of H^+^ by other ions.^36−38^ The 1-PA titrates Brønsted sites in H-form zeolites and can provide reasonable estimates of residual Brønsted sites in metal-exchanged zeolites because desorption peaks associated with 1-PA on the metal cations shift to higher or lower temperatures. This titration also detects framework atoms that might give rise to weaker acid sites and their departure from the framework.^43^ An example analysis for the three catalysts supported on LTL is shown in Figure 1a.

The low-temperature peaks (A and B) are associated with Lewis acid interactions with the 1-PA. The 1-PA decomposes to propylene and NH_3_ in a Hofmann elimination reaction on Brønsted acid sites (H^+^), and the products desorb at 350–440 °C (peak C). Stronger Lewis sites are also sometimes found, thought to be associated with extraframework Al (peak D in Figure 1a). It is evident that for PtSn/K-L and Pt/K-L, there are no observable Brønsted or strong Lewis acid sites, meaning all ion-exchange sites are occupied by either K^+^, Sn^2+^, or Pt^2+^. The 1-PA titration data for the other catalysts can be found in Figure S4.

XPS was used to probe the electronic states of Pt and Sn in the zeolite LTL catalysts. Pt 4f_7/2_ spectra were observed at binding energies of 70.8 and 70.7 eV for Pt/Ba-K-L and PtSn/K-L, respectively, which suggest the Pt is mostly in its metallic state, the 4f_7/2_ and 4f_5/2_ peaks usually centered around 70.8–71.1 and 74.1–74.4 eV (Figure 1b,c),^42,44,45^ with Pt^2+^ at 74.0 and Pt^4+^ at 74.9 in the oxides.^45,46^ The lower binding energy of Pt with Sn, relative to Pt alone, is an indication of electron donation to Pt resulting from the interaction between Pt and Sn.^42^ The same phenomenon was observed for the Pt_5/2_ peaks, which gave binding energies of 74.5 eV for Pt/Ba-K-L and 74.0 eV for PtSn/K-L, again indicating mainly metallic Pt.^42,45,47^ Linear combination fits of Pt(0) foil and PtO (Figure S5) regressed 79 and 72% of the Pt as Pt(0) for Pt/Ba-K-L and PtSn/K-L, respectively. The binding energy of Sn was observed at 486.9 eV (Figure 1d), indicating the presence of Sn^2+^ or Sn^4+^.^47^ This is consistent with the possibility that the Sn species donate electron density to Pt.

### Catalyst Performance

**Figure 2:**
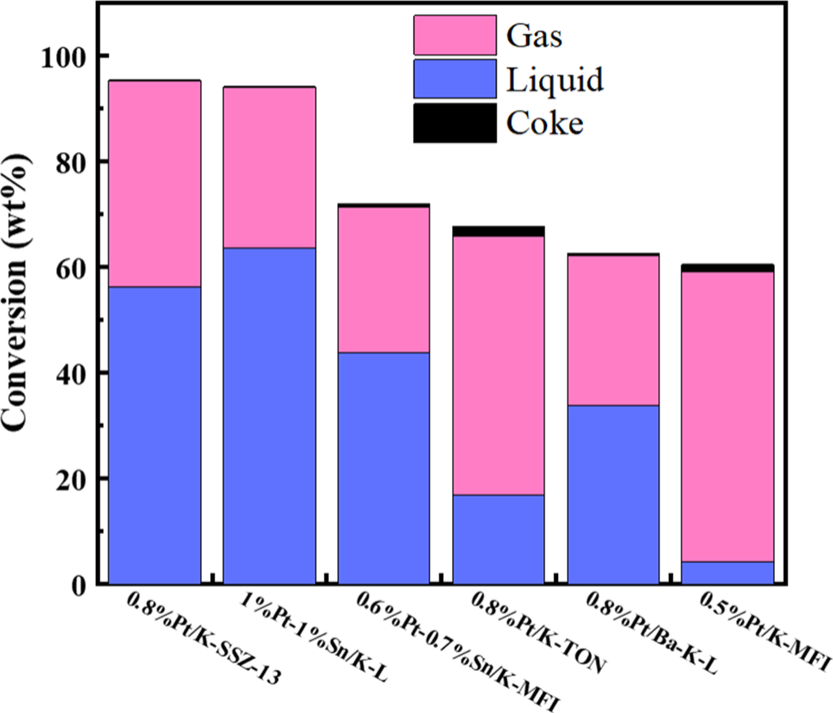
LDPE depolymerization with IH, 54 mT (500 A, 375 °C) input for 2 h at autogenous pressure. Liquid, gas, and coke conversions are on a weight basis.

**Figure 3:**
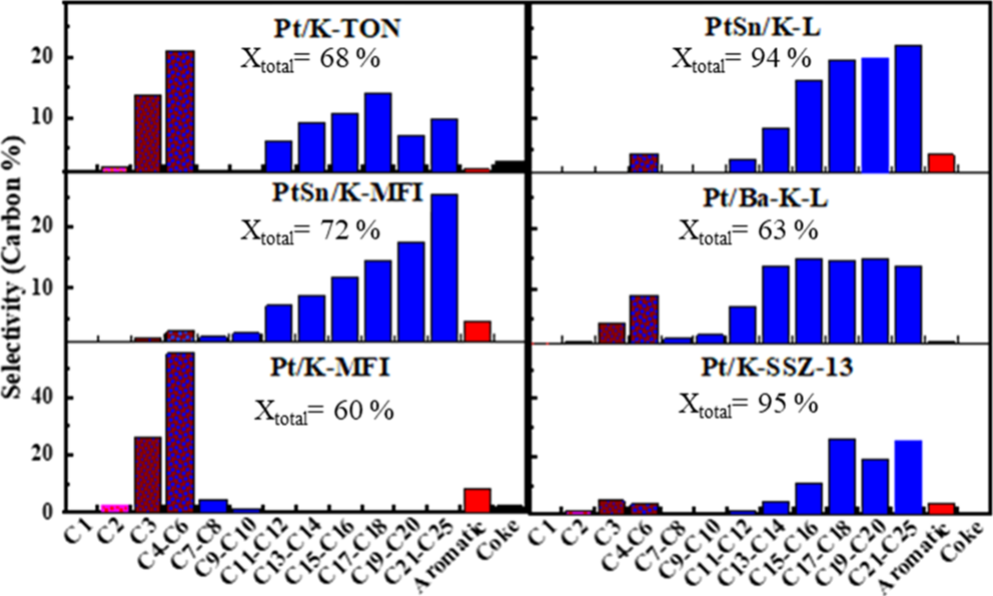
Product distributions on carbon mol % basis for LDPE depolymerization at 500 A, 2 h.

The results of depolymerization experiments using commercial LDPE are shown in Figure 2 with more detailed tabular data in Table S1 and the mass balances for the 500 A data in Table S2. The TPOs used for coke determination are in Figure S10. All the catalysts were reduced before the reaction. PtSn/K-L and Pt/K-SSZ-13 are the most active catalysts (ca. 95 wt % total conversion) for IH depolymerization at 375 °C for 2 h (Figure 2). PtSn/K-L is also best at making liquid products (∼64 wt % liquid yield) at 375 °C, coupled with the lowest production of coke. When Pt was supported on LTL without Sn, the liquid yield dropped by almost half, with no significant change in gas yield but an increase in coke formation (Table S1). Comparing results from Pt-containing L, TON, SSZ-13, and MFI zeolites, it is evident that the dispersion of the Pt plays little to no role in the overall activity, while the catalyst morphology and the presence of Sn do. Pt on MFI and TON, known to have smaller pore sizes of 0.51–0.57 and 0.46–0.57 nm, respectively, were selective to lighter products (Figure 3).

**Figure 4:**
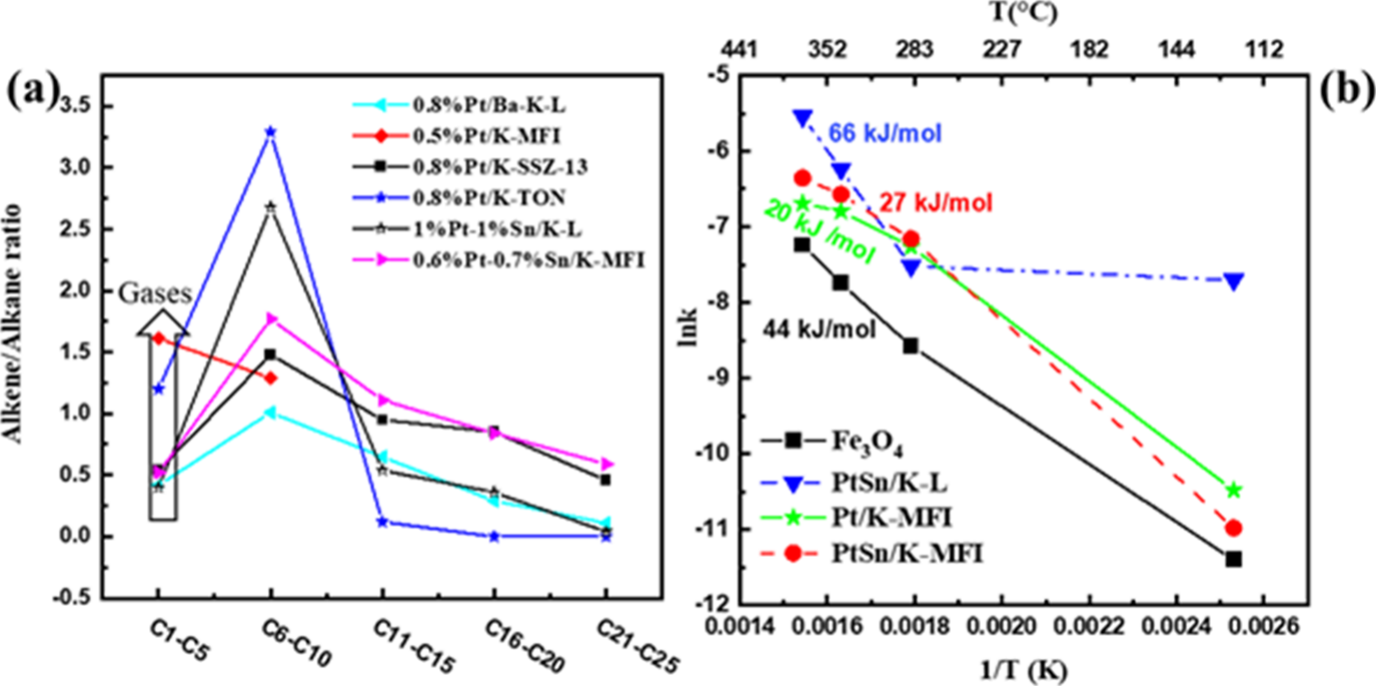
(a) Alkene-to-alkane ratio of the product distributions. (b) Activation energy for IH depolymerization of LDPE with Fe_3_O_4_ and Pt-based LTL and MFI catalysts. The activation energy *E*_a_ was calculated at temperatures of 285 °C and higher. The *R*^2^ values of these fits were 1, 0.99, 0.97, and 0.99 for PtSn/K-L, Fe_3_O_4_, Pt/K-MFI, and PtSn/K-MFI, respectively.

But the addition of Sn to either MFI or LTL zeolites both enhances catalytic activity and shifts the selectivity more to the higher molecular weight (and probably higher value) liquid products (Figure 3). These effects must be due to the Pt–Sn interaction. The moderation of cracking activity is one of the salient features of properly made Pt–Sn catalysts. Overall, Pt/K-MFI generated the highest alkene-to-alkane ratio, probably due to the formation of lighter products (Figure 4a) so more cracking events. Even though different catalysts generated different proportions of alkenes to alkanes, there is a similar trend of decreasing alkene/alkane ratio with respect to increasing carbon number for the liquid products (>C5). Therefore, the type of catalyst and the process conditions can be optimized to maximize the production of either alkenes or alkanes at the expense of a reduction in average molecular weight. This behavior is consistent with the lack of significant (or in some cases, any) aromatics or coke production.

From Table 2, it is seen that the activity of PtSn/K-L is reduced by more than 50% between 400 and 300 A, or roughly 340 to 285 °C surface temperature. For thermal depolymerization, we found essentially no activity for zeolite catalysts until ∼350 °C, and even then the conversions were low.^33^ Pt/K-MFI was used for additional conventional thermal (“Joule heating”) experiments at the same conditions as the IH reactions (Table S3 and Figure S6). Although the product distribution was geared toward light gases for both thermal and IH, the conversion was much higher for IH than thermal. The IH-driven rate constant was found to be ca. 13 times higher than the thermal one (Table S3 and Figure S6). It would appear as if the IH-driven process is more effective in heat transfer to the catalyst, with some activity even taking place at temperatures near the LDPE melting point. It is also true that the Fe_3_O_4_ catalyzes some depolymerization, as shown in Figure S7. However, the activity is relatively low, with almost no activity at 200 A (122 °C). Notably, the product distributions for the Pt-only catalysts look very different from the product distributions for Fe_3_O_4_ alone (which are skewed toward higher molecular weight products).^33^ The distributions for PtSn catalysts are also skewed toward higher molecular weight alkenes/alkanes but at much higher polymer conversions. This suggests that when a zeolite is mixed with Fe_3_O_4_, it is the zeolite doing most of the catalysis, even though the Fe_3_O_4_ by itself is slightly active.

**Table 2:** Temperature-Dependent Rate Data for PtSn/K-L, *X* = LDPE Conversion (wt %)

*T* (°C)	gas *X* (wt %)	liquid *X* (wt %)	coke *X* (wt %)	total *X* (wt %)	*k* (s^–1^)
122	6.3	19.4	2	27.7	4.50 × 10^–4^
285	8.1	20.9	3.3	32.3	5.40 × 10^–4^
340	21.1	54.1	0.2	75.4	1.90 × 10^–3^
375	30.5	63.5	0.1	94.1	3.90 × 10^–3^

PtSn/K-L, Pt/K-MFI, PtSn/K-MFI, and pure Fe_3_O_4_ were then used to depolymerize commercial LDPE over a range of IH inputs, giving four different surface temperatures (Figure 4b). For a zeolite to polymer wt ratio of 0.1, the rate constants (*k*) were calculated assuming a first-order isothermal batch reactor mass balance (eq S2), first-order in polymer concentration only, and these results can be found in Table 2 (PtSn/K-L) and more fully in Table S4. These rate constants can be compared on a first-order basis to other catalytic depolymerizations employing conventional thermal heating. For example, the *k* in Table 2 at 285 °C surface temperature is more than 20 times that for a hydrogenolysis/aromatization process that does not use H_2_ at 280 °C (Table 3).^9^ It is even comparable to polyolefin depolymerization processes that do use H_2_, greater than the derived *k*’s for catalytic processes making alkanes at 250^8^ and 200 °C,^48^ while slightly less than a *k* for a different catalyst making lubricants at 300 °C,^12^ all in Table 3. Obtaining this high a reaction rate and still a relatively narrow product distribution, without the use of any added H_2_, shows that the combination of IH with Pt–Sn zeolites is a powerful one for LDPE depolymerization.

**Table 3:** Reference Rate Constant Data for PE Depolymerization

catalyst	*T* (°C)	H_2_*P* (bar)	*k* (s^–1^)	products
1.5 wt % Pt/Al_2_O_3_	280	0	2.04 × 10^–5^	alkylaromatics^9^
5 wt % Ru/C	250	30	3.20 × 10^–4^	C8–C45 *n*-alkanes^8^
11 wt % Pt–SrTiO_3_	300	11.7	6.06 × 10^–4^	lubricants^12^
0.5 wt % Pt/15 wt % WO_3_/ZrO_2_ + HY	250	30	3.69 × 10^–3^	fuels^11^
5 wt % Ru/FAU	200	30	4.71 × 10^–4^	C5–C33 alkanes^48^

The Arrhenius equation was then used to estimate the observed activation energies (Figure 4b). The relationship between ln *k* and 1/*T* is linear only at higher temperatures. The activation energy *E*_a_ was calculated only at temperatures of 285 °C and higher. The low values of these observed *E*_a_’s, when compared to the intrinsic hydrocracking activation energy of ∼150 kJ/mol for an alkane,^49^ suggest significant diffusion limitations are present in this process. Clearly, the process is even more highly diffusion limited at below 285 °C, possibly entering into a different diffusional regime for some or all of the catalysts.

It has been shown that surface diffusion of planar zig-zag conformers allows long-chain alkanes/alkenes to penetrate the pores of zeolite to some extent, thus controlling the size and shape of the cracked products.^33,50−52^ Therefore, rates of diffusion would depend not only on conventional pore diffusion but also on the rate at which the polymer chains can adopt the correct conformation to even enter the pores or to interpenetrate zeolite crystals, and on the diffusion rate of both chains and products through the liquid boundary layer. These phenomena are highly complex and interactive, and a full discussion is beyond the scope of this paper. However, we note that the observed *E*_a_’s for self-diffusivities and mutual diffusivities of differing (by branching, molecular weight, or both) PEs and of longer chain alkanes such as dodecane in PEs vary from 16 to 28 kJ/mol based on PFG-NMR measurements.^53−56^ The *E*_a_’s in Figure 4b are either near these values or between them and 75 kJ/mol, suggesting that in some cases diffusion through the liquid boundary layer is the limiting resistance, while in others the serial combination of this resistance and the pore diffusion resistance determines the observed *E*_a_.

### Characterization of Used Catalysts and Discussion

**Figure 5:**
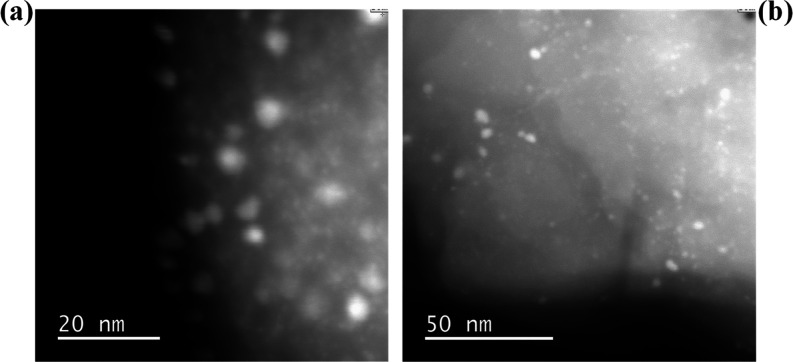
HRTEM images of: (a) fresh PtSn/K-L catalyst; (b) spent PtSn/K-L catalyst after depolymerization of LDPE at 375 °C for 2 h.

TEM images of fresh and used PtSn/K-L are shown in Figure 5. The average particle size of the active metals (Pt and/or Sn) for the fresh catalyst is 3.6 ± 1.0 nm. Significant sintering of metal particles has been observed for PtSn/K-L at 873 K when used for isobutane dehydrogenation.^17^ Such sintering was also observed for this catalyst but to a much smaller extent. The average particle size of the used PtSn/K-L is 3.7 ± 1.2 nm (Figure S8). This suggests the reusability of the catalyst is possible.

XRD analysis on both used and unused Fe_3_O_4_ revealed that the Fe_3_O_4_ did not reduce to Fe after reaction at either 122 or 375 °C (Figure S9). However, some iron carbide (Fe_3_C, cementite phase) was detected on the used catalyst. The Fe_3_C peaks at 2Θ 37.6 and 40.6° were more pronounced after the higher temperature reaction.

**Figure 6:**
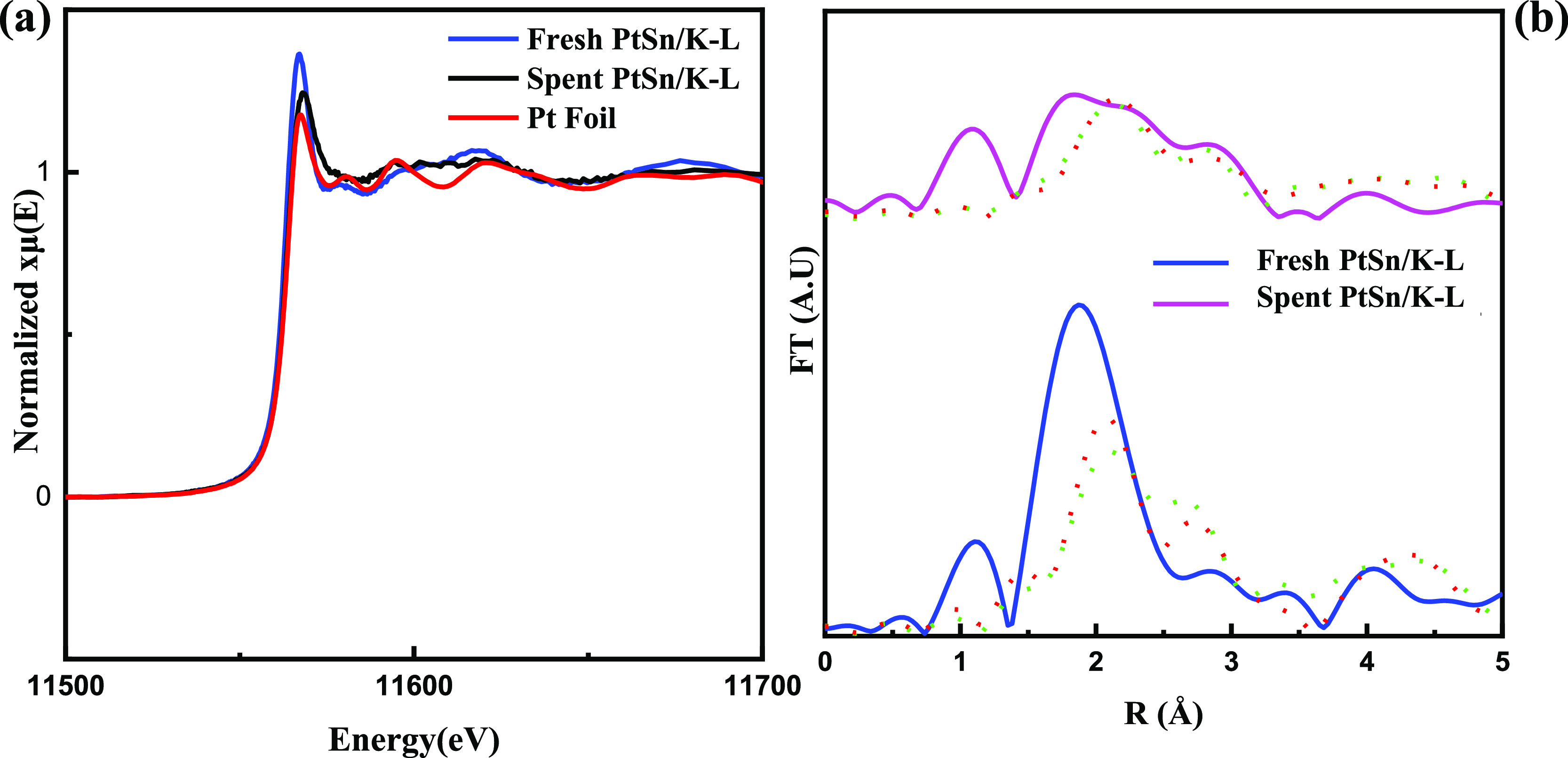
(a) Pt L_III_ XANES spectra for Pt–Sn/K-L catalysts (b) Fourier transformed XAFS spectra for fresh and used PtSn/K-L. Green and red dotted lines represent fits to bulk Pt and Pt_3_Sn, respectively.

We used Pt L_III_ XANES to probe the oxidation state of the Pt–Sn/K-L catalyst before and after the reaction (Figure 6a). The slight shift of the edge position to a lower energy compared to the Pt foil standard shows that the Pt is electron rich, probably due to some electron transfer from Sn to Pt,^14,18,57^ as was also observed in the XPS spectra (Figure 1b,c). It is evident that the Pt is mostly in the metallic [Pt(0)] state both before and after the reaction. Metallic Pt within and on the external surfaces of zeolite crystals is known to be active for both alkane dehydrogenation and alkene cracking and would therefore be preferred to Pt^2+^ for depolymerization reactions. However, the decrease in white line intensity for the used catalyst suggests some sintering has taken place, in agreement with the HRTEM data.

The X-ray absorption fine structure (XAFS) spectra support the hypothesis that there is a Pt–Sn interaction in both unused and used catalysts (Figure 6b). The theoretically generated FT XAFS spectra for metallic Pt and the common bulk alloys of Pt with Sn (Pt_3_Sn, PtSn, and Pt_2_Sn_3_)^58^ were fitted to both unused and used PtSn/K-L FT XAFS. The regression parameters for these are given in Table S5. Of the bulk alloys, Pt_3_Sn fit the experimental spectra best, slightly better than bulk Pt. However, the imperfect fits for all of these suggest there is no bulk alloy structure, i.e., no large clusters of any single compound. The fit of the experimental data to the Pt_3_Sn structure improved significantly for the used catalyst (the *R*-factor changed from 0.40 unused to 0.27 used). This could be due to additional Pt–Sn alloying occurring during the reaction or the aforementioned ripening to larger metal particles. Additional Pt–Sn alloying would play a role in limiting the hydrocracking to lighter hydrocarbons, which was observed for all of the Pt–Sn catalysts (Figure 3).

The observed relative activities of the zeolite catalysts can be explained in part by the balance between diffusional and intrinsic confinement effects. For light alkanes in zeolites, acid-catalyzed cracking and dehydrogenation reactions strongly depend upon confinement effects.^59^ As pore space decreases, −Δ*S*_ads_ of the alkane increases, and the configurational bias Monte-Carlo-computed Δ*H*_app_^⧧^ (apparent activation enthalpy, accounting for adsorption effects) for central and terminal cracking decreases linearly, while Δ*H*_app_^⧧^ for dehydrogenation decreases exponentially. The range of the latter, even for 10-MR zeotypes only, is large, ∼100 kJ/mol. By these metrics, one might expect an activity order for depolymerization independent of diffusional effects of SSZ-13 > TON > MFI > L. It turned out that Pt/K-SSZ-13 was quite active. It is considered a small-pore zeolite (8, 6, and 4 ring pore openings), but it also has ∼7.37 Å diameter supercages. As seen in Table 1, it also has a high surface area and pore volume, which would also favor high activity.

However, the diffusive path lengths differ for some of these zeolites. From the *t*-plot analyses, the computed nanocrystal sizes are in the 20–25 nm range for Pt/K-TON but in the 4–5 nm range for the others. So, while Pt/K-TON might be expected to be a more generally active catalyst and more selective to higher molecular weight products on the basis of confinement effects alone, the larger crystal size for this catalyst depressed the overall depolymerization activity and led to higher molecular weight products. The smaller crystal sizes and larger pore sizes for both of the PtSn catalysts mean easier diffusion of reactants and products, and, as seen, these are highly diffusion-limited reactions.

Examining previous work on PtSn/K-L, while it is not the most common zeolite-based dehydrogenation catalyst, it is known to be quite active. Hill and co-workers measured isobutane dehydrogenation TOFs (surface Pt basis, 600 °C, 2:1 H_2_/isobutane) in the ratio 3.3:1.3:1 for PtSn/K-L:Pt/K-L:PtSn/K-SiO_2_.^17^ These rates are in line with our findings here.

## Conclusions

In summary, Pt(0), either alone or when combined with Sn, when supported on zeolites, showed excellent activity for the depolymerization of LDPE without the use of H_2_. This was especially so when the energy input is provided by RF-IH and was demonstrated by comparisons both to our catalysts and to others’ when heated conventionally (“Joule heating”). The IH depolymerizations showed high concentrations of alkene/alkane hydrocarbons with a narrow product distribution that could be tuned between light gases and gasoline to diesel-range hydrocarbons based on the zeolite structure. PtSn/K-L and Pt/K-SSZ-13 were the most active catalysts, giving a total LDPE conversion of about 95 wt % for IH depolymerization of LDPE at 375 °C for 2 h without added H_2_. The addition of Sn as a promoter was found to increase activity with a preference for heavier products but to reduce coke formation. All of these catalysts generated low amounts of methane, aromatics, and coke.

Pt/K-MFI was the most selective catalyst to light gases and generated the highest alkene-to-alkane ratio. A decreasing alkene/alkane ratio with an increasing carbon number for the liquid products (>C5) was observed for all the catalysts. Kinetics analysis showed that the depolymerization process is diffusion limited across the temperature range of 122–375 °C.

The catalyst functioned with Pt particles present in the zero-valent state, whether with or without Sn. Minimal metal particle ripening was observed after the reaction.

## Supporting Information Available

Reactor schematic, synthesis and XRD of SSZ-13 and Fe_3_O_4_, dispersion calculation, algorithm to convert RGA data to gas-phase compositions, N_2_ adsorption isotherms of the catalysts, 1-PA acid site titrations, reactor mass balances, IH and thermal depolymerization conversion, rate and selectivity data, XPS linear combination fitting results, particle size distributions from HRTEM for PtSn/K-L, EXAFS fitting results for PtSn/K-L, TPO analyses, and reactor mass balances (PDF)
