# Supplementary material for: Zeolite Supported Pt for Depolymerization of Polyethylene by Induction Heating
Source: Ind Eng Chem Res. 2023 May 24;62(22):8635–43. doi: 10.1021/acs.iecr.2c04568 (PMC10251740; doi:10.1021/acs.iecr.2c04568)
Supplement: Supplementary file 1 — ie2c04568_si_001.pdf [file ie2c04568_si_001.pdf]

Supporting Information

Zeolite Supported Pt for Depolymerization of Polyethylene by Induction Heating

Bernard Whajah[1], Joseph N. Heil[2], Cameron L. Roman[1], James A. Dorman[1,*], and Kerry M. Dooley[1,*]

[1]Cain Department of Chemical Engineering, Louisiana State University, Baton Rouge, Louisiana 70803, United States

[2]Department of Chemistry and Physics, LeTourneau University, Longview, Texas 75602, United States

*dooley@lsu.edu

*jadorman@gmail.com

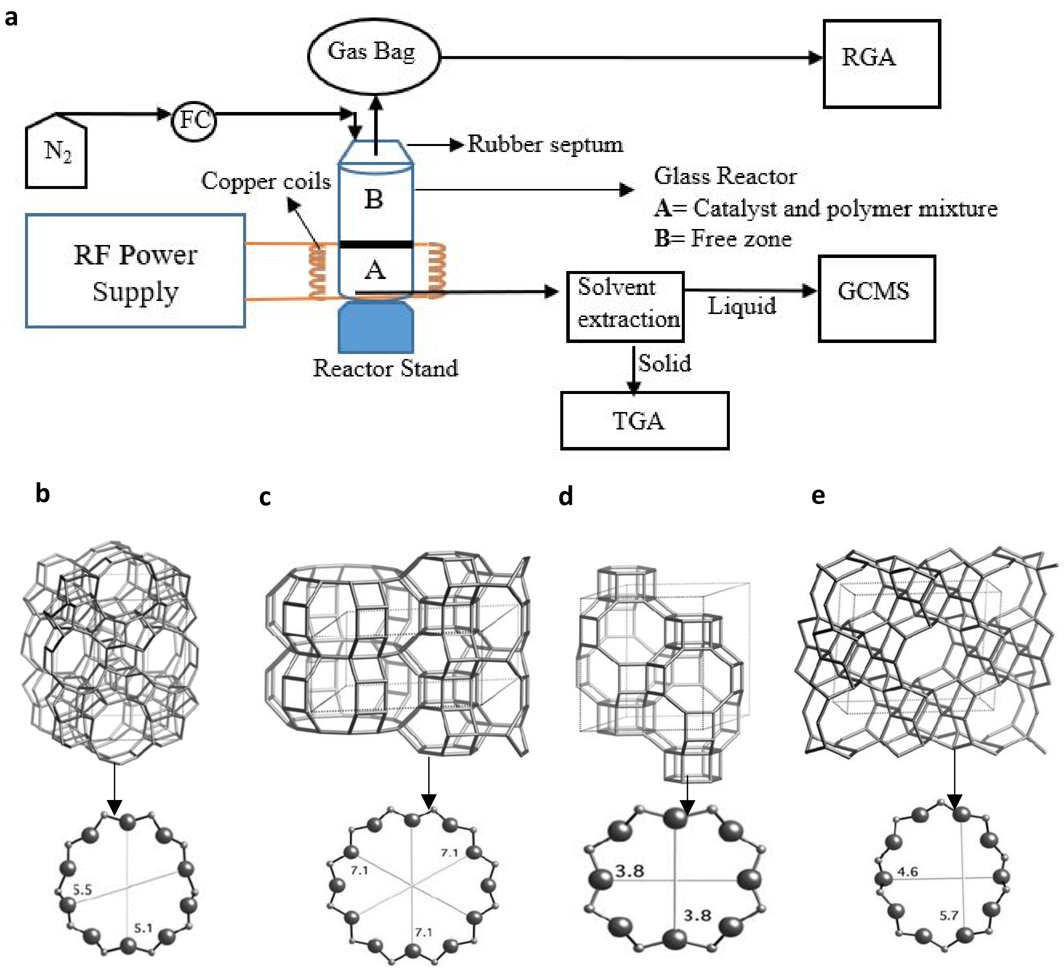

Figure S1. (**a**) Schematic of depolymerization reactor system; (**b**) MFI zeolite structure, 10 ring elliptical pores 5.5 × 5.1 Å (equivalent spherical diameters), viewed along [010] direction; (**c**) LTL zeolite structure, viewed along [001] direction, 12 ring 7.1 Å circular pores; (**d**) CHA (SSZ-13) zeolite structure, 8 ring with 3.8 Å openings, viewed normal to [001]; (**e**) TON zeolite structure, 10 ring with 4.6 × 5.1 Å pore diameters, viewed along [001]

Zeolites Used. The MFI used here was Zeolyst lot 5534G-1597-94 and the Si/Al ratio was measured by 1-PA TPD.[1] The L-zeolite was commercial Linde Type L (LTL). The TON was synthesized[1] using a tetraethylenepentamine (TEPA) template (Pfaltz and Bauer, 99.5%) in a modification of the methods of Ernst et al.[2] and Pellegrino et al.[3] Further synthesis details and the characterization are given in ref. 1. The XRD showed all the expected peaks and no impurities. The SSZ-13 was synthesized starting from a boron-BEA.[4] This borosilicate was converted to a B-SSZ-13 using a N,N,N-trimethyl-1-adamantammonium hydroxide (TMAD) template. The synthesis ratios (per 1 g B-BEA) were: 1 M aqueous TMAD, 2.5 mL; 1 M aqueous NH_3, 1.7 mL; additional water, 9.8 mL. After mixing all ingredients other than B-BEA, the B-BEA and the mixture were added to an autoclave, and heated at 150 °C for 2 d at 10-20 rpm. The final product gave an XRD pattern very similar to the SSZ-13 of Zones (Fig. S2).[5]

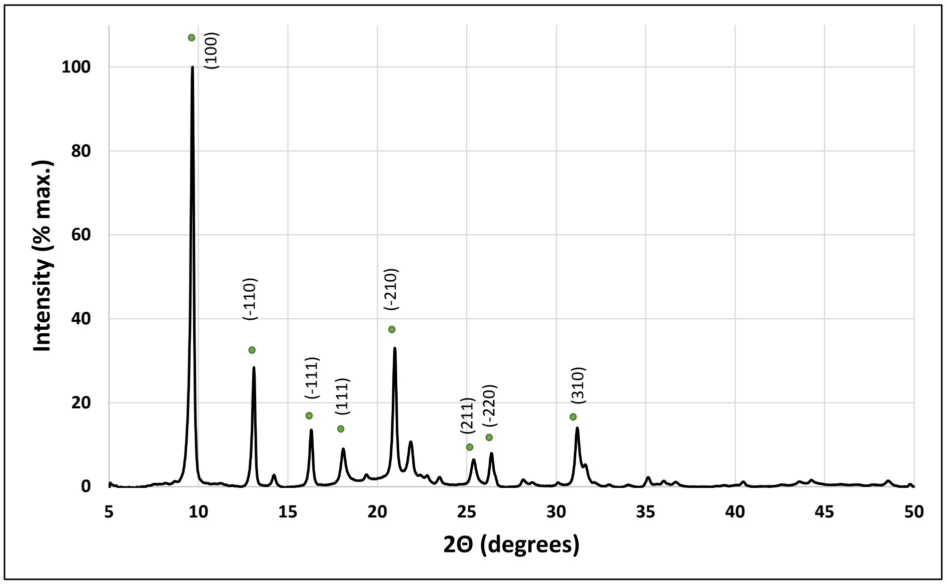

Figure S2. XRD pattern of K-SSZ-13

Equation S1.

$$\%\boldsymbol{D} = \frac{\boldsymbol{Vc} * \boldsymbol{m.w} * \boldsymbol{n} * \mathbf{100}}{\boldsymbol{ms} * \boldsymbol{Vg} * \boldsymbol{M}}$$

%D = % Dispersion

Vc = Volume chemisorbed, cm^3

m.w = molecular weight of active metal

n = stoichiometry factor

ms = mass of sample, g

Vg = molar volume gas at STP = 22414 cm^3/mol

M = % metal loading

Algorithm to convert RGA data to gas-phase compositions

ΔP (Pressure change from background prior to injection) α **Y** (concentration)

ΔP32 (Change in P at m/e = 32) = $\mathbf{Y_{O2}}$ **x** $\mathbf{IF_{O2}}$

where IF_{O2} is the ionization factor for oxygen

Oxygen is used as the internal standard with $IF_{O2} = 1$. Relative to O_2, IF_{N2}= 1.07, IF_{H2O} = 0.959.

These three were the background (BKG) components.

$$\Sigma(\text{BKG}) = \Sigma\,(\,Y_{N2} + Y_{O2} + Y_{H2O}\,) = \Sigma\,[(\,\Delta P32)\,(79/21) + (\,\Delta P32) + (\,\Delta P18\,/\,0.959\,)]$$

For air calibration,

$$\frac{\Sigma(\text{BKG}) \; - \Sigma(\text{BKG,0})}{\Sigma(\text{BKG,0})} = \frac{N_{Air}}{N_0}$$

BKG,0 = BKG composition in the RGA prior to air injection

N_0 = Moles of BKG composition in the RGA prior to air injection

In other calibrations,

$$N = (\,\frac{\Sigma(\text{BKG})}{\Sigma(\text{BKG,0})}\,)\,N_0$$

Where N represents mols of BKG comps in RGA at BKG conditions

$$\frac{\Sigma\,[(\,\Delta P32)\,(79/21) + (\,\Delta P32) + (\,\Delta P18\,/\,0.959\,)]}{\Sigma(\text{BKG})} = \frac{X}{N}$$

X = Mols BKG components entering with the calibration compound

For methane,

$$\frac{1 / (IF_{CH4})[\Sigma \Delta P_{16}] - \Sigma(\Delta P32)(0.218) - \Sigma[(\Delta 18)(0.009)/(0.959)]}{\Sigma[(\Delta P32)(79/21) + (\Delta P32) + (\Delta P18 / 0.959)]} = \frac{\Psi}{X}$$

For other components,

$$\frac{1 / (IF_j)[\Sigma \Delta P_j]}{\Sigma[(\Delta P32)(79/21) + (\Delta P32) + (\Delta P18 / 0.959)]} = \frac{\Psi}{X}$$

Ψ = Mols of calibration comp. j

Fragmentation patterns of the compounds used, from the NIST database, m/e(% relative intensity).[6]

O_2 = 32(100) ; 16 (21.8) N_2 = 28(100) ; 29 (0.70) H_2O = 16(0.90) ; 18(100)

C_2H_4 = 27(62.3); 28 (100) CH_4 = 15 (88.7); 16(100)

C_3H_6 = 15(5.5); 27(38.7); 28(0.14); 41(100); 42(70.3); 44(0.10)

Let Y_E = Ethane Y_2 = Ethylene Y_P = Propane Y_3 = Propene

Y_B = Butane Y_4 = 1-Butene Y_Z = Pentane Y_5 = Pentene

For pentene and pentane respectively,

$\Delta P70 = (IF_5)(Y_5)$ $\Delta P72 = (IF_z)(Y_z)$

For ethylene,

$$\Delta P27 = (IF_2)(Y_2) + (IF_E)(Y_E)(0.332/0.262) + (IF_4)(Y_4)(0.251/0.388) + (IF_B)(Y_B)(0.387/0.122) + (IF_P)(Y_P)(0.419/0.274) + (IF_3)(Y_3)(0.387/0.703) + (IF_5)(Y_5)(0.182/0.394)$$

Relative intensity of the 27 ion in the frag. pattern of propane

Relative intensity of the molecular ion (44) in the frag. pattern of propane

The molar concentration (Y_2) of ethylene was calculated from the ethylene equation above. Similar equations were used for the other components. The molar composition with respect to time was averaged or integrated and then normalized to determine the gas selectivity, which was then combined with total gas conversion to quantify the gaseous product.

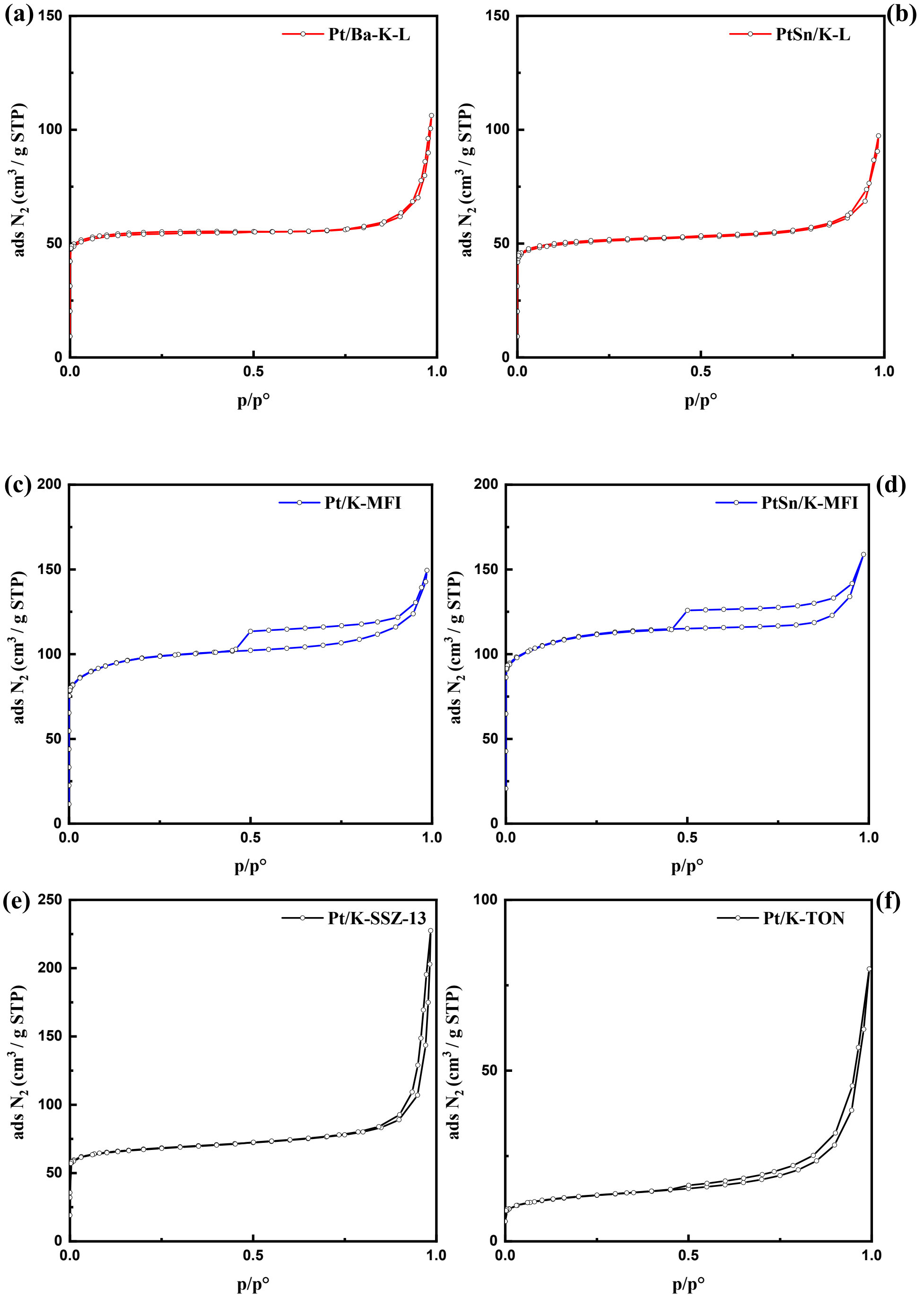

Figure S3. N_2 adsorption Isotherms for a) Pt/Ba-K-L, b) PtSn/K-L, c) Pt/K-MFI d) PtSn/K-MFI, e) Pt/K-SSZ-13, f) Pt/TON

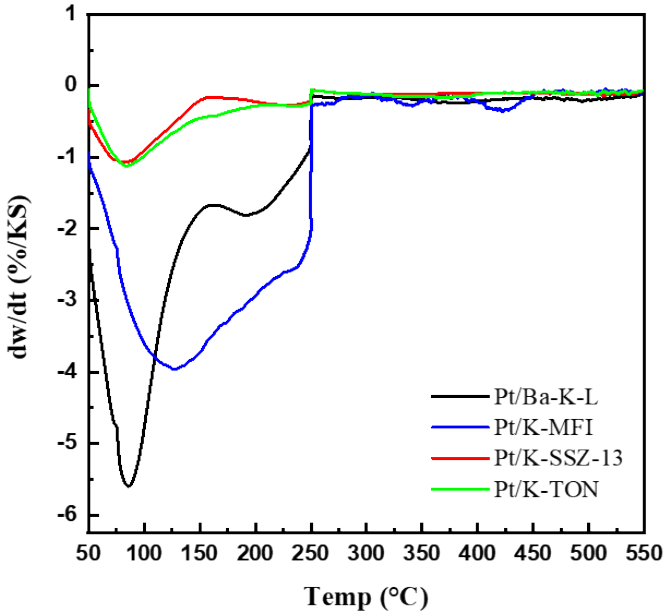

Figure S4. Desorption of 1-propylamine (1-PA) from the four zeolites containing Pt but no Sn. Samples saturated with 1-PA at 50° C prior to TPD.

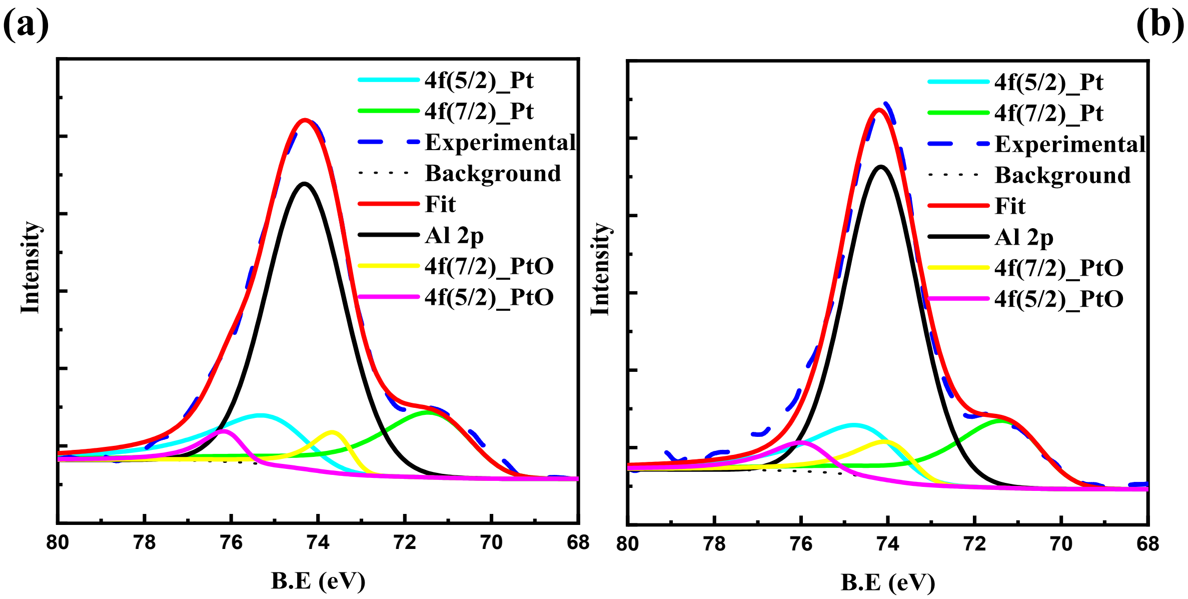

Figure S5. Linear combination fits of XPS data for (a) Pt/Ba-K-L and (b) PtSn/K-L to Pt(0) foil and PtO standards.

Table S1. Conversions of LDPE for all catalysts at all conditions tested

Catalyst	Surface.T(˚C)	Gas X(wt.%)	Liquid X(wt.%)	Coke X(wt.%)	Total X(wt.%)	Mol% H_2 / Gas X
0.8%Pt/Ba-K-L	375	28.5	33.7	0.4	62.6	12.1
1%Pt-1%Sn/K-L	375	30.5	63.5	0.1	94.1	35.0
1%Pt-1%Sn/K-L	340	21.1	54.1	0.2	75.4	-
1%Pt-1%Sn/K-L	285	8.1	20.9	3.4	32.4	-
1%Pt-1%Sn/K-L	122	6.3	19.4	2.1	27.8	-
0.5%Pt/K-MFI	420	80.9	1.5	0.3	82.7	-
0.5%Pt/K-MFI	375	54.8	4.3	1.3	60.4	11.2
0.5%Pt/K-MFI	340	50.5	4.8	-	55.3	-
0.5%Pt/K-MFI	285	54.8	6.4	-	39.6	-
0.5%Pt/K-MFI	122	1.9	0.3	-	2.2	-
0.6%Pt-0.7%Sn/K-MFI	375	27.5	43.8	0.7	72.0	56.4
0.6%Pt-0.7%Sn/K-MFI	340	34.9	37.1	-	63.5	-
0.6%Pt-0.7%Sn/K-MFI	285	14.6	28.4	-	42.9	-
0.6%Pt-0.7%Sn/K-MFI	122	0.7	0.5	-	1.2	-
0.8%Pt/K-TON	375	48.9	16.8	2.0	67.7	8.5
0.8%Pt/K-SSZ-13	375	38.9	56.2	0.2	95.3	2.4
Fe_3O_4	375	12.8	27.4	-	40.2	-
Fe_3O_4	340	7.3	19.4	-	26.7	-
Fe_3O_4	285	5.5	12.7	-	18.2	-
Fe_3O_4	122	0.3	0.5	-	0.8	-

Table S2. Mass balances for LDPE depolymerization at 500A, 2 h.

Catalyst	Total m (P_{in}) (g)	m (G_{out}) (g)	m (L_{out}) (g)	m (C_{out}+UP_{out}) (g)	Mass Balance (% out/in)
Pt/K-MFI	1	0.55	0.04	0.43	102
PtSn/K-MFI	1	0.26	0.44	0.29	99
Pt/Ba-K-L	1	0.29	0.34	0.38	101
PtSn/K-L	1	0.31	0.64	0.05	100
Pt/K-TON	1	0.49	0.17	0.35	101
Pt/K-SSZ-13	1	0.39	0.56	0.06	101

P_{in} is the mass of LDPE charged to the reactor. G_{out} and L_{out} represent the masses of gas and liquid products measured during and after reaction and extraction. C_{out} + UP_{out} refer to coke plus unreacted LDPE polymer.

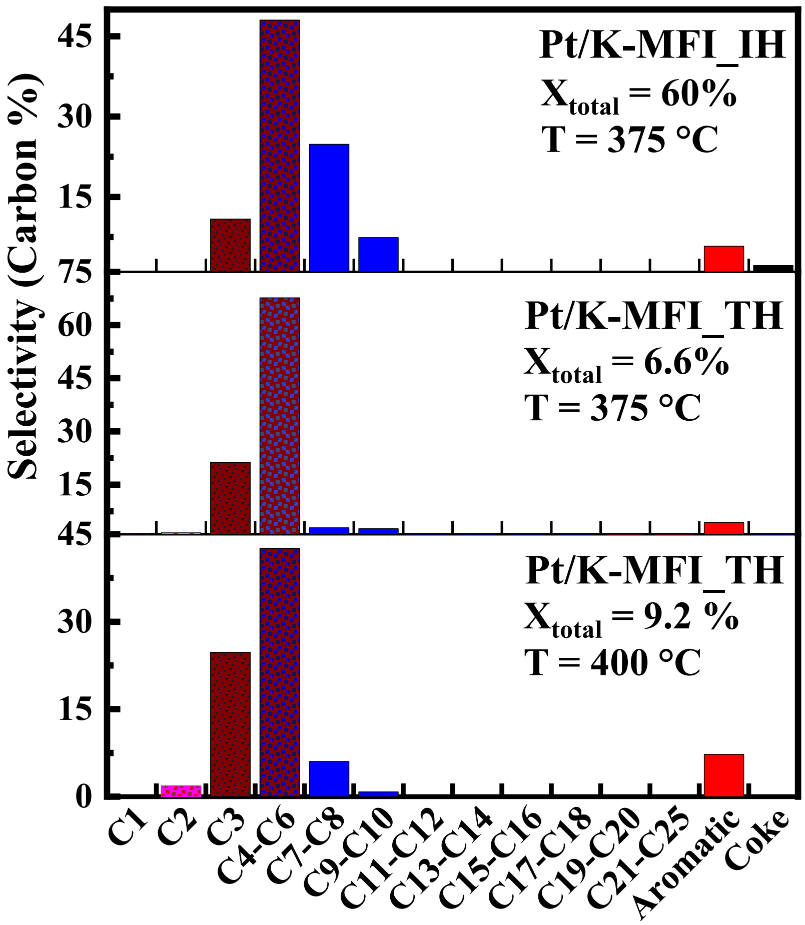

Figure S6. Product distributions on carbon mol% basis for LDPE depolymerization using thermal (TH) and induction heating (IH). Apart from the mode of heat transfer, same experimental conditions described in the methods section, were used. Coke analysis was not done for the thermal runs due to the very low total conversion.

Table S3. Thermal (TH) and IH conversion and rate data for Pt/K-MFI.

T (°C)	Gas X (wt.%)	Liquid X (wt.%)	Total X (wt.%)	Mol% H_2/Gas X	k (s^{-1})
375_IH	54.8	4.3	59.1	11.2	1.24E-03
375_TH	5.4	1.2	6.6	2.3	9.48E-05
400_TH	6.4	2.8	9.2	3.6	1.34E-04

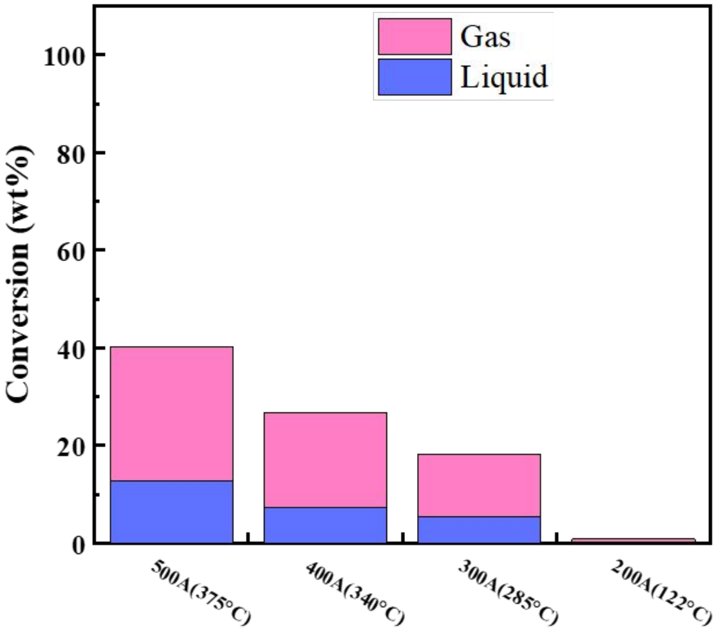

Figure S7. LDPE depolymerization catalyzed by Fe_3O_4 nanoparticles with RF-IH input for 2 h, at autogenous pressure. Liquid and gas conversions are on a weight basis.

Equation S2.

$$-ln(1-f) = \frac{k\,C_0}{N_o} V_c\, t = k\,\varepsilon_c\, t$$

This is the batch reactor mass balance for a catalytic reactor. Here f is the fractional conversion of polymer to all products, C_o the initial concentration of polymer on a molar basis, N_o the initial mols of polymer, V_c the catalyst volume, and ε_c the catalyst/polymer volume ratio. The rate constant has units [fluid vol/(cat vol x time)], or s^{-1}.

Table S4. Temperature-dependent rate data

Catalyst	Surface.T(˚C)	k (s^{-1})
1%Pt-1%Sn/K-L	375	3.92E-03
1%Pt-1%Sn/K-L	340	1.95E-03
1%Pt-1%Sn/K-L	285	5.43E-04
1%Pt-1%Sn/K-L	122	4.51E-04
0.5%Pt/K-MFI	375	1.24E-03
0.5%Pt/K-MFI	340	1.12E-03
0.5%Pt/K-MFI	285	7.00E-04
0.5%Pt/K-MFI	122	2.81E-05
0.6%Pt-0.7%Sn/K-MFI	375	1.73E-03
0.6%Pt-0.7%Sn/K-MFI	340	1.40E-03
0.6%Pt-0.7%Sn/K-MFI	285	7.78E-04
0.6%Pt-0.7%Sn/K-MFI	122	1.70E-05
Fe_3O_4	375	7.14E-04
Fe_3O_4	340	4.31E-04
Fe_3O_4	285	1.89E-04
Fe_3O_4	122	1.12E-05

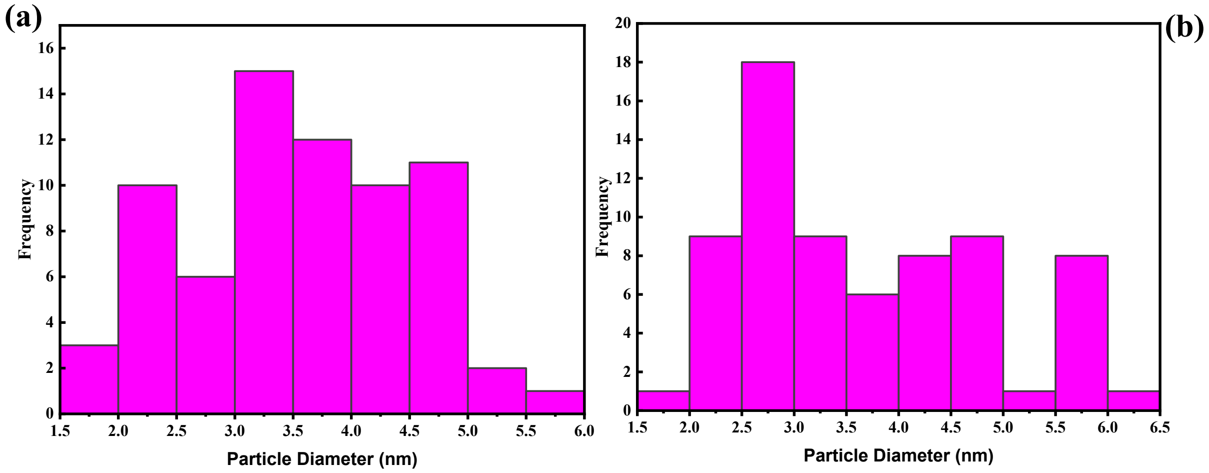

Figure S8. Pt/Sn particle size distribution from HRTEM images for a) Fresh PtSn/K-L and b) Spent PtSn/K-L

Table S5. EXAFS fitting results for fresh and spent PtSn/K-L.

Sample	Path	N	σ^2 (Å^2)	R (Å)	R-factor
Fresh PtSn/K-L	Pt-Pt	8 ± 0.034	0.00928 ± 0.00297	2.58852 ± 0.01953	0.40
	Pt-Sn	4 ± 6.95	0.02167 ± 0.02337	2.37226 ± 0.02788	0.40
	Pt-Pt[a]	12 ± 0.03081	0.01007 ± 0.00248	2.60249 ± 0.01917	0.43
Spent PtSn/K-L	Pt-Pt	8 ± 0.00654	0.01200 ± 0.01331	2.64560 ± 0.02898	0.27
	Pt-Sn	4 ± 0.22697	0.0200 ± 0.01383	2.90686 ± 0.09662	0.27
	Pt-Pt[a]	12 ± 0.00639	0.01521 ± 0.00187	2.66059 ± 0.02758	0.27

[a] This Pt-Pt first-shell fit is from the regression of the experimental data to the Feff computation for bulk metallic Pt, while the earlier fits are from regression to the Feff computation for Pt_3Sn. N is the coordination number, σ^2 the Debye–Waller factor, and R is the Pt-Pt or Pt–Sn distance. The fits to PtSn and Pt_2Sn_3 structures are not included due to their very large R-factors.

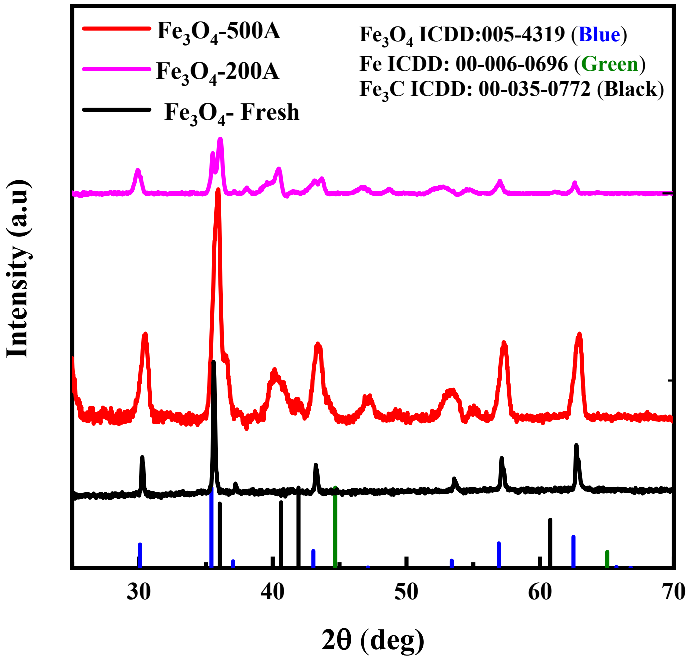

Figure S9. XRD pattern of fresh Fe_3O_4 and used Fe_3O_4 at 500A (Red) and 200A (Magenta). The intensity of the fresh Fe_3O_4 is scaled down 3x.

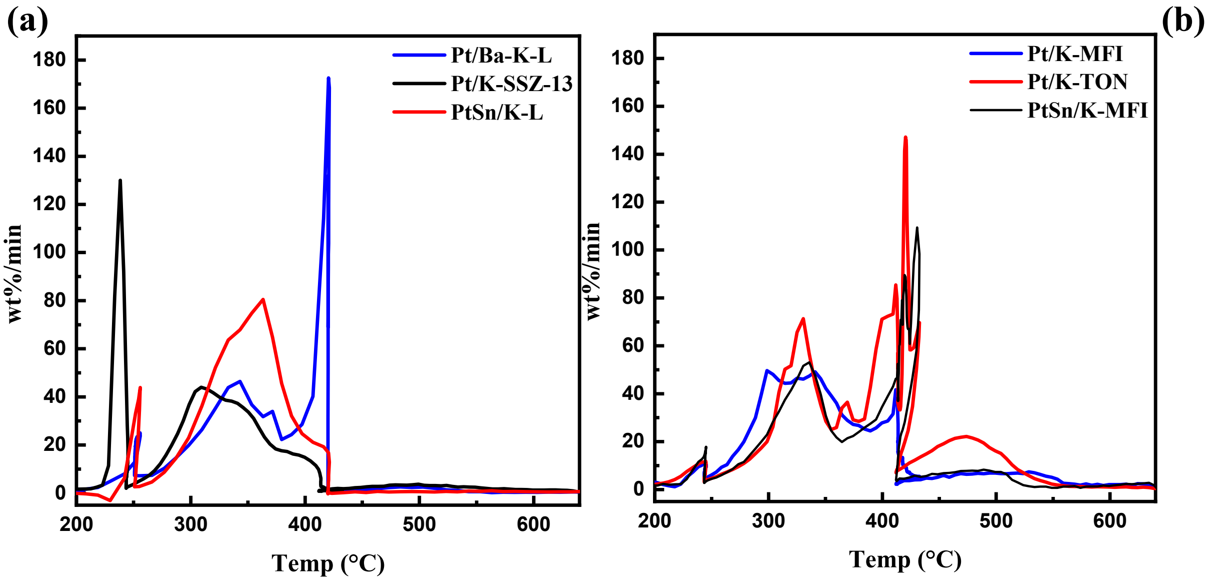

Figure S10. Coke analysis by TPO, for catalysts (a) Pt/Ba-K-L, Pt/K-SSZ-13, PtSn/K-L (b) Pt/K-MFI, Pt/K-TON, PtSn/K-MFI, after LDPE depolymerization at 500A, 2 h, followed by extraction of liquid products. The presence of coke is seen from the peaks at greater than 420 °C.

Literature Cited

1. Hart, V. I. Metal (Ga, In, Sn)-loaded zeolite dehydrogenation catalysts. M.S., Louisiana St. Univ., Baton Rouge, LA, 1998.

2. Ernst, S.; Kokotailo, G. T.; Kumar, R.; Weitkamp, J., Shape Selective Catalysis in Zeolites ZSM-22 and ZSM-23: Influence of Pore Shapes on Reaction Selectivities. *Proc. 9th Intl. Cong. Catal., Calgary* **1988,** *1*, 388-395.

3. Pellegrino, C.; Aiello, R.; Gabelica, Z., Stabilizing Role of Linear Polyamines as Pore Fillers During the Crystallization of Zeolite NU-10. In *Zeolite Synthesis*, Occelli, M. L.; Robson, H. E., Eds. ACS: Washington, D.C., 1989; pp 161-175.

4. Zones, S. I.; Nakagawa, Y., Boron-beta zeolite hydrothermal conversions: the influence of template structure and of boron concentration and source. *Micro. Mater.* **1994,** *2*, 543-555.

5. Zones, S. I. Zeolite SSZ-13 and its Method of Preparation USP 4,544,538, 1985.

6. Wallace, W. E., "Mass Spectra" by NIST Mass Spectrometry Data Center. In *NIST Chemistry WebBook, NIST Standard Reference Database Number 69*, Linstrom, P. J.; Mallard, W. G., Eds. National Institute of Standards and Technology: Gaithersburg, MD, 2022.
